# At-home, self-sampling of the skin microbiome: development of an unsupervised sampling approach

**DOI:** 10.1099/acmi.0.000991.v3

**Published:** 2025-08-08

**Authors:** J. Leng, J. Tyson-Carr, S. Adams, M. Scott, A. Thomas, T. Giesbrecht, N. Fallon, B. Murphy, M. Hoptroff, C. Roberts, S. Paterson

**Affiliations:** 1 Institute of Infection, Veterinary and Ecological Sciences, University of Liverpool, Liverpool, L69 7ZB, UK; 2 Department of Psychology, Institute of Population Health, University of Liverpool, Liverpool, L69 7ZB, UK; 3 Unilever Research and Development, Port Sunlight, Bebington, Wirral, CH63 3JW, UK

**Keywords:** 16S rRNA sequencing, remote sampling, skin microbiome

## Abstract

Large-scale skin microbiome studies are often restricted due to the need for participants to visit a research centre to have their skin swabbed by a trained individual. If samples taken by participants at home returned high-quality data, similar to that generated from samples taken by trained experts under controlled conditions, it would provide the potential for studies to have larger cohorts, include participants from multiple locations and facilitate longitudinal sample collection. Here, we describe the development of a novel unsupervised skin microbiome sample collection method and compare the data quality with that of supervised, in-lab sample collection. We enrolled 57 participants to collect skin swabs from their axillae, forearms, cheeks and scalps. Initially, samples were collected in our research centre under strict supervision by a trained expert. Participants then collected swabs from the same body sites 24 h later, unsupervised, at home, which they returned to the research centre within 3–5 days. All samples then underwent bacterial DNA extraction and 16S rRNA gene sequencing. Yield of extracted bacterial DNA was different depending on body site, with the dry swabs from the forearm producing the lowest amount. There were no significant differences in alpha and beta-diversities between supervised and unsupervised sampling methods, regardless of body site. Taxonomic analysis of bacterial genera also did not differ for axilla, cheek or scalp. Our data suggest that self-sampling skin microbiome methods can produce data that are comparable to samples collected under the supervision of a trained expert in lab settings. These findings should encourage the scalability of future research and allow for greater representative population diversity in genomic and microbiome research.

## Data Summary

The sequencing data were generated by Illumina sequencing of the V1–V2 region of the 16S rRNA amplicon and are available on the National Centre for Biotechnolgy Information(NCBI) Sequence Read Archive (SRA) under Bioproject PRJNA1189034, where supervised samples were uploaded as SUB14875555 and unsupervised samples as SUB14883787.For SUB14875555 accession numbers for the samples are SRR31433672 - SRR31433714. For SUB14883787 sample accession numbers are SRR31478029 - SRR31478177. 

## Introduction

The human skin microbiome comprises the bacteria, fungi and viruses found on the human stratum corneum and within the integuments, such as the phyllis sebaceous. They perform important functions on the skin, such as protecting the body against pathogens and contributing to the immune response [1,2]. Changes in the skin microbiome have been documented in several skin diseases. For example, an increase in the relative abundance of *Staphylococcus aureus* and a reduction in *Staphylococcus epidermidis* and *Corynebacterium* spp. is seen in individuals suffering from atopic dermatitis when compared with healthy controls [3]. Several papers have reported an increase in several staphylococcal species (including *S. aureus*) in people suffering from psoriasis, as well as decreased *S. epidermidis*, *Cutibacterium acnes* and *Cutibacterium granulosum* compared to healthy individuals [4,5]. Non-disease state conditions have also been linked to differences in the skin microbiome. Individuals suffering from dandruff were found to have higher levels of *Staphylococcus* spp. and fungal species belonging to the *Malassezia* genus [6]. These studies highlight the importance of the skin microbiome to overall body health and how changes may be linked to the onset of disease.

To adequately power studies examining the profile of, or the effect of a skin product on, the skin microbiome, large sample sizes over extended periods of time are needed to account for inter-individual variation within the skin microbiome of a population [7]. Another challenge when conducting skin microbiome studies is that the study population often excludes parts of the human population including those unwilling or unable to take skin microbiome samples. There is a gap in both human genomics and human microbiome research where individuals from Africa, South America and Asia are vastly underrepresented in most studies [8,9]. Barriers to including these groups of people in more microbiome studies include fear of exploitation and distrust of scientific researchers [10]. Currently, most skin microbiome studies collect samples from Caucasian participants from Western countries. However, differences in both the bacterial diversity and composition of the skin microbiome between ethnic groups have been reported [11]. This would suggest that results from studies analysing only the skin microbiome from Caucasian participants are only directly relatable to a portion of the human population. A practical hurdle in achieving a representative, large sample size for a study is that sampling often requires participants to visit a research site where a trained person can collect a sample of the skin microbiome. Thus, the development of high-quality remote (unsupervised) sampling methods of the skin microbiome could improve scalability, as well as allow for greater representative population diversity in genomic and microbiome research.

Self-sampling microbiome techniques would allow for the expansion in size and location of human microbiome studies. However, this has not been validated and has the potential to introduce variation into microbiome data due to contamination and differing storage. When microbiome samples are collected at research centres, they are often immediately frozen, and remote sampling would not allow for this. The development and validation of a simple tape stripping method to sample the skin microbiome has previously been reported, with the aim of quality skin microbiome samples, irrespective of the participant’s skill level [12]. Methods have also been published validating the use of at-home, unsupervised collection of samples to analyse other human microbiomes. For example, flexibility in the collection site of human stool samples for human gut microbiome studies has been required to generate adequate sample numbers and data for meaningful studies. It has been successfully validated in several studies where stool samples were returned by post and then analysed using 16S rRNA gene amplicon sequencing [13,14]. These studies show that there is the potential for successful analysis of the human skin microbiome utilizing samples taken by participants at home.

Here, we developed an unsupervised, at-home, self-sampling approach of the human skin microbiome at four body sites (axilla, forearm, scalp and cheek). These four body sites were chosen to cover a range of sebaceous activity and occlusion levels. We aimed to assess the feasibility of unsupervised skin microbiome sampling methods by comparing data quality to that of samples collected under the supervision of a trained expert at a research centre. Samples were collected by dry swabbing of the skin to make self-sampling more practical for participants. Bacterial DNA extracts from skin swabs were analysed using 16S rRNA gene amplicon sequencing to generate data on bacterial community diversity as well as taxonomic composition. This has the potential to remove a key barrier to the enrolment of sizable numbers of participants, reduce sample bias by enabling participants who would ordinarily not be able or willing to take part, increase the diversity of the backgrounds of people sampled to be more representative of the whole human population and reduce the reliance on trained individuals for sample collection.

## Methods

### Development of the self-sample method for unsupervised in-home skin microbiome sampling

At-home, non-supervised skin microbiome methods were developed at Unilever Research and Development Laboratories in Port Sunlight (Wirral, UK). This utilized skin swabbing for ease of sample collection, and no immediate refrigeration or freezing of swabs was necessary, as samples were returned by post. This method was adjusted for use in this study to include the collection of scalp samples, no immediate refrigeration/freezing of swabs, the exclusion of buffer to dampen the swabs before swabbing and samples were returned by participants within 3–5 days (detailed instructions given to participants are in Item S1, available in the online Supplementary Material).

### Study participants

A total of 29 women and 27 men were enrolled in the study, with a mean age of 63.21±12.06 years (Item S2). Participants were required to be over 18 years of age and willing to collect skin microbiome samples unsupervised (at home). Exclusion criteria included the presence of a cold or flu, any active skin conditions on any part of the body, taking any prescribed antibiotics and being pregnant or having given birth in the last 6 months. Participants were asked not to shower or apply deodorant or antiperspirant to their underarms 6 h prior to sampling (for both supervised and unsupervised sampling). The experimental procedures were approved by the Unilever Research and Development Port Sunlight ethics committee. All participants gave written informed consent in accordance with the Declaration of Helsinki. Participants were reimbursed for their time and travel expenses.

### Skin microbiome sampling

During supervised collection of skin microbiome samples in the research centre, participants were instructed to swab a 2 cm area of skin on their forearm, axilla, face (on one cheek) and scalp using a new sterile dry, flocked swab (VWR) for each body site. Dry flocked swabs were chosen as they have previously been reported to generate skin microbiome samples that are of comparable quality [12]. Instructions on how to collect a swab sample were explained by a trained microbiologist. After the sample was collected, the end of the swab was snapped off by the participant and placed into a sterile, empty cryovial for storage. Samples taken in the research centre were transferred to a −25 °C freezer at the end of the day. All participants were given an at-home sampling pack which contained four swabs, four cryovials, study information and instructions on how to collect a skin swab sample (see Item S1) and a swabbing checklist to ensure all four body sites were sampled.

Unsupervised samples were collected by participants using the same sampling technique 24 h later at home. The side of the body that was sampled under supervision for the forearm, axilla and face was noted on take-home material. Participants were asked to swab the same side when taking the at-home sample. Samples were stored at room temperature and returned to the research centre 3–5 days after the sample was collected. Upon arrival at the laboratory, all samples were stored at −25 °C until thawed for DNA extraction.

Samples were defrosted before DNA extraction and left to thaw at room temperature for 30 min. DNA was extracted using Extracta Reagent (Quantabio, USA) and lysing matrix B tubes (MP Biomedicals, UK). The head of the swabs was severed into the lysing matrix tubes, and 400 µl of the extraction reagent was added. Tubes were subjected to bead beating for 1 min, followed by centrifuging for 1 min. The supernatant was removed and mixed by pipetting with an equal volume of stabilization buffer from the Extracta Kit.

### Quantitative PCR 

The bacterial DNA concentration of all samples was generated using a quantitative PCR (qPCR) assay for total bacteria developed by QIAGEN (Germany). PCR assays consisted of 0.5 µl of primer and probes mix with a final concentration of 400 nM and 200 nM, respectively, 5.5 µl of UCP Probe PCR Kit (QIAGEN, Germany) and 4.5 µl of template DNA. Samples and standards were amplified using the following parameters: 95 °C for 2 min, then 40 cycles of 95 °C for 45 s and 60 °C for 10 s, using a QIAGEN Rotor-Gene Q (QIAGEN, Germany). The amount of the target in each sample was determined by comparing the cyle threshold (Ct) value to the calibration curve and quoted in copies per microlitre.

### 16S rRNA gene amplicon sequencing

Illumina sequencing of the V1–V2 region of the 16S rRNA gene was conducted as previously outlined in Murphy *et al*. [15]. DNA extracts, along with positive and negative controls, were submitted to the Centre of Genomic Research, University of Liverpool for sequencing. The first PCR targeted the V1–V2 hypervariable region of the 16S rRNA gene using the primers U28F: ACACTCTTTCCCTACACGACGCTCTTCCGATCTNNNNNAGAGTTTGATCMTGGCTCAG and U338R: GTGACTGGAGTTCAGACGTGTGCTCTTCCGATCTTGCTGCCTCCCGTAGGAGT. PCR reactions consisted of 0.5 µl (10 µM) of each primer, 10 µl of HotStar Taq Plus Mastermix (QIAGEN), 5 µl of template DNA and 4.5 µl of molecular grade water (Ambion, Thermo Fisher). Thermocycling conditions for this PCR were as follows: 95 °C for 5 min and then 10 cycles of 94 °C for 45 s, 65 °C for 30 s and 72 °C for 60 s, with a final extension of 10 min at 72 °C. A second round of PCR to incorporate Illumina indexes (i5 and i7). The primers used for this were N501f: AATGATACGGCGACCACCGAGATCTACAC*TAGATCGC*ACACTCTTTCCCTACACGACGCTC and N701r: CAAGCAGAAGACGGCATACGAGAT*TCGCCTTA*GTGACTGGAGTTCAGACGTGTGCTC. Second round PCR assay reactions consisted of 0.5 l (10 µM) of each primer, 10 µl of 2 × KAPA Mastermix (Roche, Switzerland) and 9 µl of purified sample from the first PCR reaction. The underlined section denotes the variable 8 bp barcode. The PCR conditions for this second PCR were as follows: 98 °C for 2 min and then 15 cycles of 95 °C for 20 s, 65 °C for 15 s, 70 °C for 30 s with a final extension of 72 °C for 5 min. Sequencing was completed on the MiSeq Illumina platform with 2×250 bp paired-end sequencing. Raw sequencing files were processed and analysed using a pipeline provided by Eagle Genomics [15]. Only samples with more than 10,000 sequencing reads were carried forward for further analyses. QIIME2 [16] artefacts (feature-table.qz, rooted-tree.qza and taxonomy.qza, along with metdata.tsv) were imported into R Studio as a phyloseq object [17] for further analysis. The R packages microbiome [18], random forest, fastANCOM [19] and ggplot2 [20] were used for analysis and visualization of the data within the phyloseq object.

## Results

### The total bacterial DNA amount in unsupervised samples did not differ from samples taken under supervision

**Fig. 1.**
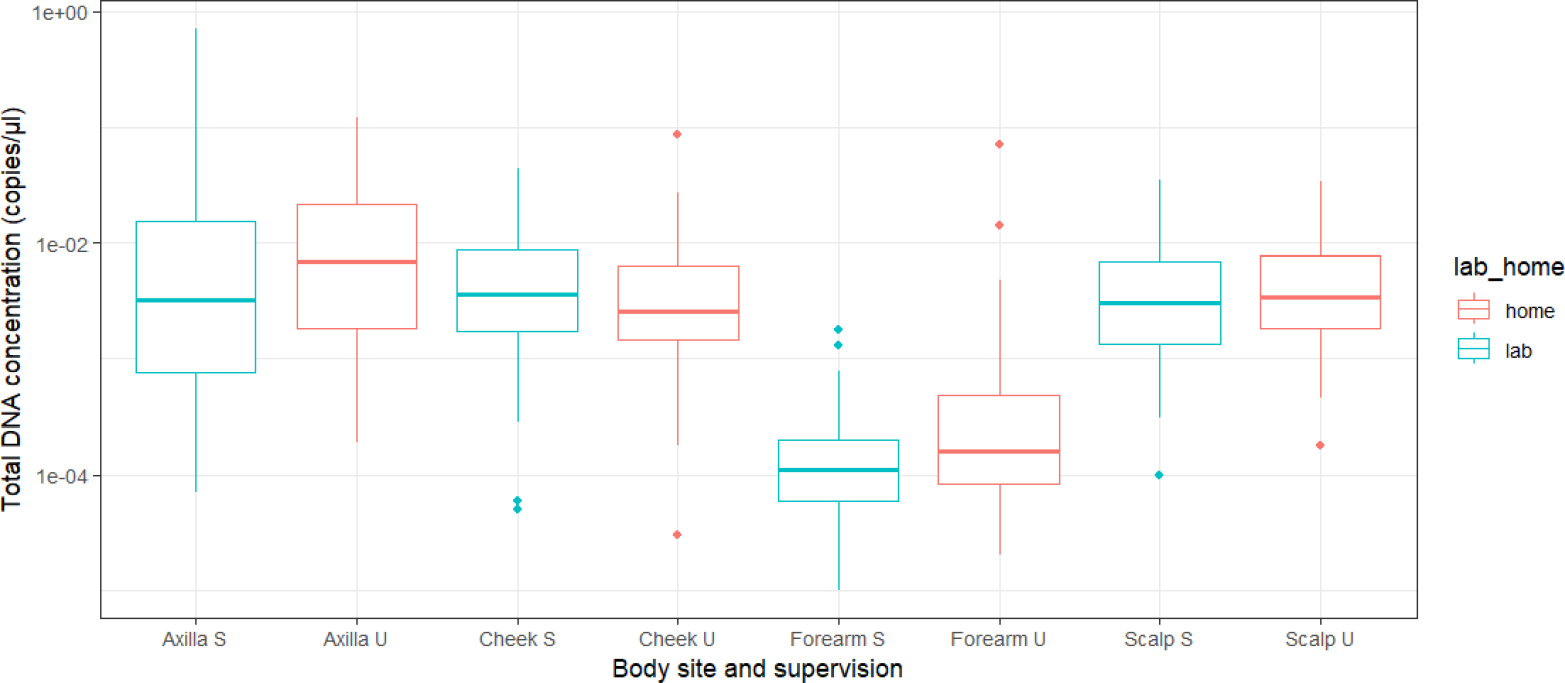
Boxplot showing bacterial DNA concentration data generated from the total bacteria qPCR assays carried out on all DNA extracts. Samples are grouped by both body site and whether samples were collected supervised (S) or unsupervised (U). There were no significant differences between samples taken of the sample body site when comparing those taken in the lab and at home (pairwise t-test, *P*>0.05).

A total of 448 skin swab samples were collected (half with supervision and half without). All samples underwent 16S qPCR to check that DNA extraction was successful and to assess whether concentrations of extracted bacterial DNA differed between samples taken under supervision and those taken unsupervised. There were no differences in total bacterial DNA load between supervised and unsupervised samples from any of the four body sites assessed (Fig. 1, paired t-test, *P*>0.05). Samples collected from the forearm had the lowest DNA concentration of the four body sites, with an average total bacterial DNA concentration across both supervised and unsupervised samples of 0.001 ng µl^−1^. Samples from the scalp and cheek had similar average total bacterial DNA concentrations of 0.005 and 0.006 ng µl^−1^, respectively, and axilla samples had the highest average total bacterial DNA concentration at 0.027 ng µl^−1^.

### The number of sequencing reads returned from samples taken unsupervised did not differ from those taken with supervision

Of the 450 samples sequenced, 87 of these did not pass initial quality control (no libraries detected after initial PCR amplification of the 16S rRNA gene V1–V2 region). These samples comprised 44 forearm samples (23 supervised and 21 unsupervised), 25 scalp samples (12 supervised and 13 unsupervised), 17 cheek samples (9 supervised and 8 unsupervised) and 1 unsupervised axilla sample. The same number of supervised and unsupervised samples failed quality control (44 and 43, respectively). There was also no difference in the number of supervised and unsupervised samples failing when splitting samples by body site. The Illumina sequencing of the V1–V2 region of the 16S rRNA gene for the remaining 363 samples returned a mean number of reads of 59,422 (range 243–307,615). Sixteen samples with fewer than 10,000 sequences were not carried forward for further analyses (Item S3). Sequencing reads returned from samples taken unsupervised did not differ from those taken under supervision from the same body site (pairwise t-test, *P*>0.05, Item S4).

### Bacterial diversity measures from unsupervised samples did not differ from those taken under supervision

**Fig. 2.**
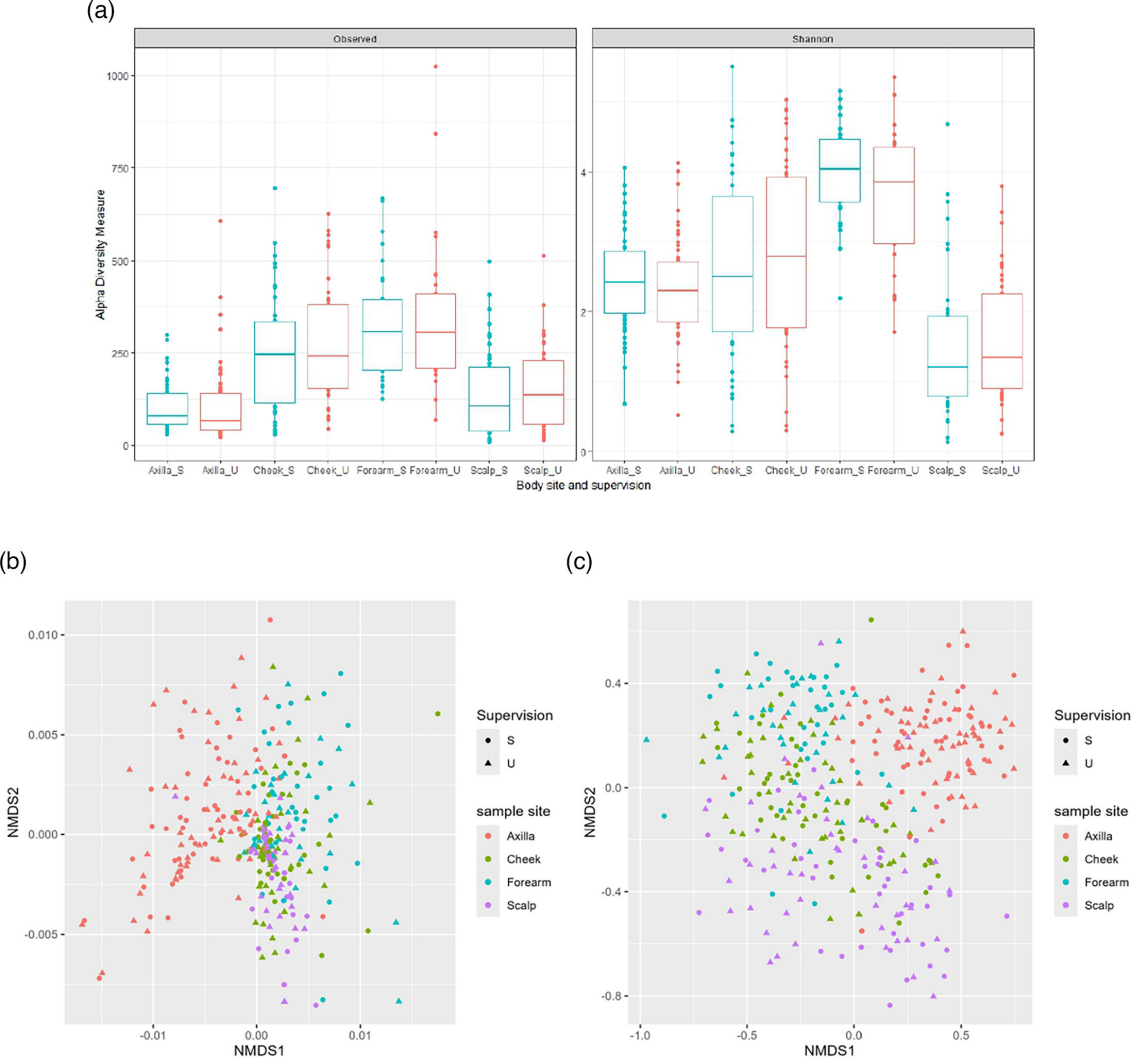
Bacterial diversity measures generated from the analysis of the 16S rRNA gene sequencing. (**a**) Alpha diversity was measured as observed and Shannon and samples were grouped by both body site and whether samples were taken under supervision (S, supervised, and U, unsupervised). Paired Wilcoxon tests found that there were no significant differences between samples taken supervised and unsupervised from the same body site (*P*>0.05). NMDS plots showing (b) weighted UniFrac and (c) Bray–Curtis distance for samples. The shape of the points indicates whether the sample was taken under supervision and the colour is the body site sampled.

After initial quality control and filtering of samples with less than 10,000 sequences, 345 samples were carried forward for further analyses. These were 111 axilla, 89 cheek, 62 forearm and 83 scalp samples (Item S3). Bacterial diversity measures were generated for all samples to compare the bacterial diversity of supervised compared to unsupervised samples at each of the four body sites. Bacterial diversity did not differ by supervision, regardless of body site using observed and Shannon measures of alpha diversity (Fig. 2a, *P*>0.05). The values for these measures of diversity for all samples analysed were plotted to compare samples taken with and without supervision (Item S3). Of the two measures of diversity, the Shannon diversity comparison showed a stronger relationship between supervised and unsupervised samples, across the four body sites (where *x*=*y* if the two are the same). Furthermore, of the four body sites, the regression line for Shannon diversity from the cheek was closest to the *x*=*y* line (equation for the best fit line was *y*=0.74*x*+0.88) and had the highest *R*^2^ value (*R*^2^=0.69).

**Fig. 3.**
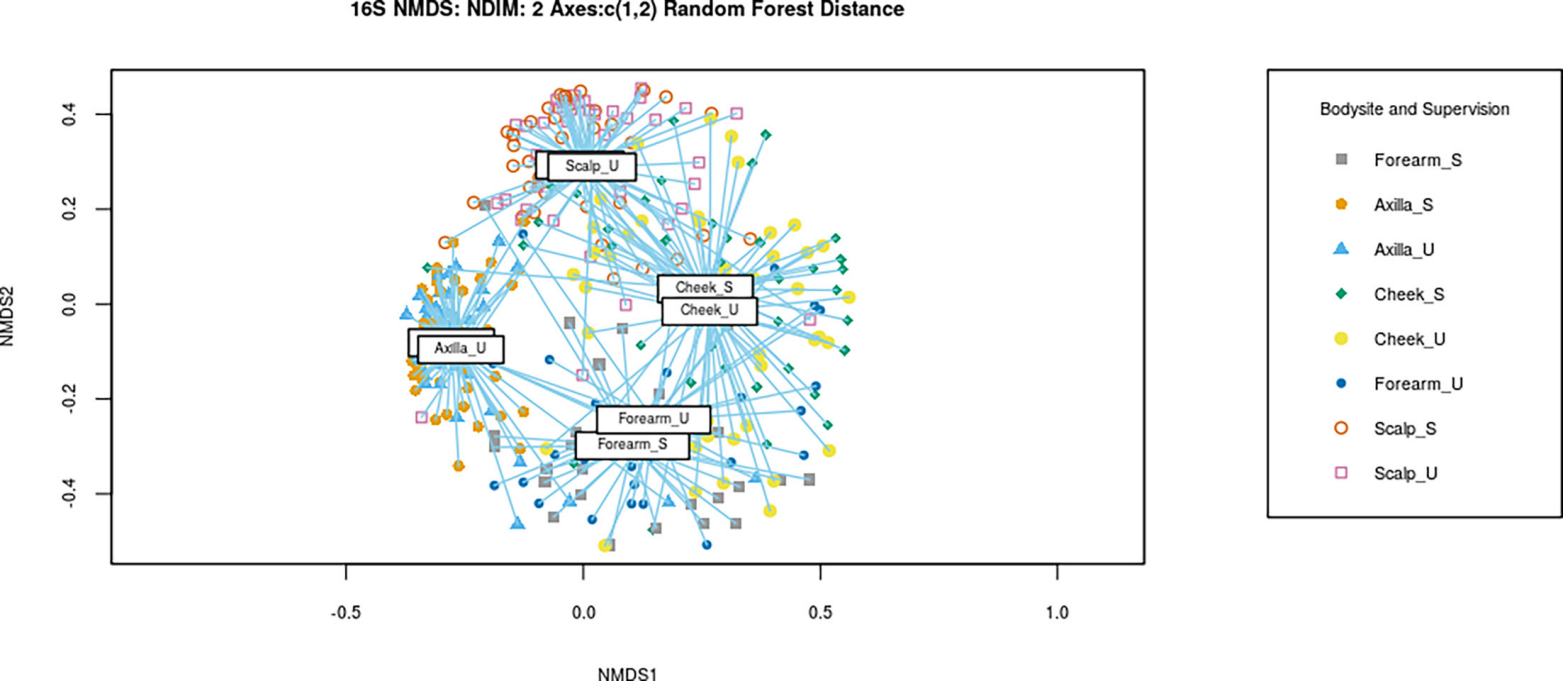
NMDS plot showing the visualization results of the random forest model. The centre of the groups (centroids) is labelled with the group name, and the lines indicate how far the samples are from their respective centroids. The centroid for ‘Scalp_S’ cannot be seen as it is underneath ‘Scalp_U’ and that for ‘Axilla_S’ is underneath ‘Axilla_U’.

Weighted UniFrac and Bray–Curtis distance matrices were generated for all samples, and non-metric multidimensional scaling (NMDS) plots were drawn using these data (Fig. 2b and c). There was no separation between the points representing samples taken supervised and unsupervised, from the same body site. Permutational multivariate ANOVA analyses were used to confirm this finding and showed that there were no significant differences between the two sampling conditions, for any of the four body sites (*P*>0.05). A random forest model was built with bacterial species relative abundance data, and an NMDS plot was drawn with the random forest distances generated (Fig. 3). Samples were grouped by both body site and supervision to assess whether samples from the same body site differ when samples were taken without supervision. The NMDS plot shows that the centre of the clusters generated for each group overlaps, indicating that they do not differ in composition. The clusters for supervised and unsupervised samples collected from the forearm were the least similar out of the four body sites, but they were still not distinct from one another within the NMDS plot.

### The relative abundance of bacterial genera identified in samples collected unsupervised did not differ from those taken under supervision

**Fig. 4.**
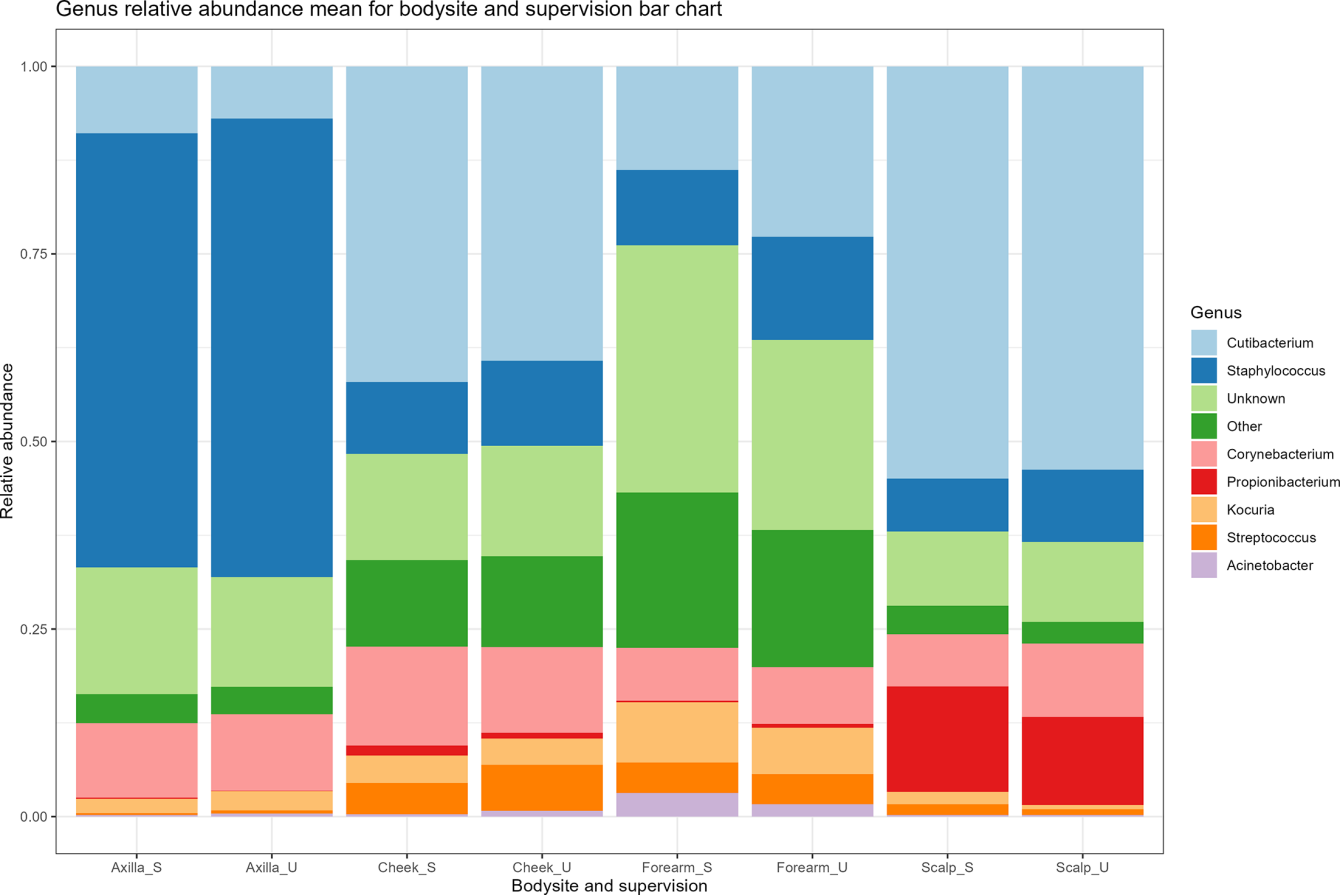
Mean relative abundance of the six most prevalent bacterial genera as a mean of samples taken supervised (S) and unsupervised (U), for each of the four body sites. All other bacterial genera identified were grouped into ‘Other’ of composition composed of lab/home and body site.

The mean relative abundance of the six most prevalent bacterial genera was calculated for each body site, for each sampling condition. This data was used to visualize these genera with the relative abundance of the remaining genera grouped into ‘Others’ (Fig. 4). *Cutibacterium* was the most abundant bacterial genus in samples taken from the cheek, scalp and forearm, and its relative abundance did not differ between the two groups of samples taken from the same body site. Similarly, the relative abundance of *Staphylococcus*, the most abundant bacterial genus in axillary samples, did not differ between sampling conditions. There were also no differences between sampling conditions in the relative abundance of the most prevalent bacterial phyla, classes, orders and families of the body sites sampled (Item S6).

### There are no significant differences in bacterial species abundance between samples supervised and unsupervised from any of the four body sites

FastANCOM was utilized to analyse the relative abundance data to identify any bacterial species that differed between samples from the same body site taken with or without supervision. This analysis was carried out separately for each of the four body sites and did not identify any species that were significantly different (*P*>0.05) and that had a false discovery rate of less than 5% (false discovery rate >0.5) (the volcano plot from the FastANCOM analysis can be found in Item S7).

## Discussion

This study demonstrated that data obtained from unsupervised sampling of the skin microbiome were comparable to that of supervised sampling. No differences in total bacterial DNA, number of sequencing reads, bacterial diversity and community composition were observed when comparing samples taken supervised and unsupervised from four body sites. This is in line with at-home/unsupervised sampling of other microbiomes, such as the faecal microbiome that is effective [13,14]. However, using dry swabs of the skin did yield relatively low amounts of bacterial DNA and so may be more likely to show contamination if a swab touched a surface other than the skin when the sample was taken. We did not see any evidence of contamination in samples collected at home by the participants of this study, indicative of non-compliance or the environment, which emphasizes the need to train participants fully before they collect samples themselves.

It was noted that samples from the forearm contained much lower concentrations of bacterial DNA compared to samples taken from other body sites. Samples of the forearm were the most abundant within the group of samples that failed sequencing quality control. The forearm is a relatively dry body site with low sebum production, but it harbours a more diverse microbial community compared to other body sites [21]. As we collected samples using dry swabs, there is the potential that samples of the skin microbiome from dry body sites may be of better quality using other methods, such as buffer scrubs. However, we chose to use dry swabbing for ease of sample collection for participants. Swabbing the skin has previously been shown to generate comparable sequencing data to other microbiome collection techniques, such as tape-stripping and skin scrapes [22,23]. The microbiome on the human arm has high diversity but low temporal stability [24]. For these reasons, dry, high-diversity sites of the skin, such as the forearm and leg, may not be as easy to sample remotely using dry swabs. There is more work needed to ascertain the best way to have participants sample such body sites for an accurate analysis of the bacterial populations found there.

The design of our study meant that samples taken unsupervised were collected by study participants 24 h after those collected under supervision, in the research centre. This may have introduced some slight variation in the bacterial communities on the skin being sampled. However, the bacterial communities that make up the skin microbiome have previously been shown to be relatively stable over time [25], and we saw no effect of sampling the skin microbiome 24 h apart during the current study. This also highlights the potential for skin microbiome samples to be collected from different participants at slightly different times, increasing the amount of flexibility within the sampling regime of a skin microbiome study.

This experiment required participants to visit a testing site for a supervised sample of the skin microbiome. This constraint of the study design will have increased compliance and accuracy of unsupervised sampling, as swabbing instructions were talked through in person. However, this could be done remotely using video calling or emails to ensure compliance. The cohort sampled during the current study had previously taken part in human microbiome studies, which may have further increased the likelihood of unsupervised sampling compliance. We were not able to assess here whether there was possible sample degradation due to the time delay between unsupervised samples being taken and frozen. This was a constraint of the study design as we aimed to make sample return easy to increase compliance. There is the possibility that samples could be returned by post or courier if unsupervised sampling was done at multiple, remote locations. There are other swabbing techniques that we did not test here, which could be utilized for at-home skin microbiome swabbing, including swabs that are submerged in gel after sample collection. The inclusion of environmental controls, such as swabs of the sampling area, would also confirm the absence of any environmental contamination within the skin microbiome data.

This study demonstrates that it is possible to have a high level of compliance and a low dropout rate during unsupervised sampling of the human skin microbiome by study participants. These samples can also be of good enough quality to produce amplicon sequencing data from analysis of extracted bacterial DNA. Relatively simple, at-home sample packs for study participants can be sent out to remote locations and samples mailed back by participants without the need for cold storage or shipping. The validation of this sampling method should encourage further human skin microbiome research with increased sampling sizes, from a wider range of locations and a more representative cross-section of the human population. Further work is needed to assess whether more complex sampling methods, such as buffer scrubs, could be utilized for at-home sampling and the potential to use fully remote sampling methods. There is also the potential for DNA extracts from such swabs to be analysed using other platforms, such as shotgun metagenomics, for a more in-depth understanding of the functionality of the bacteria that comprise the human skin microbiome.

## Supplementary material

10.1099/acmi.0.000991.v3 Uncited Supplementary Material 1.

## Abbreviations

NMDS: non-metric multidimensional scaling
qPCR: quantitative PCR
