# Supplementary material for: At-home, self-sampling of the skin microbiome: development of an unsupervised sampling approach
Source: Access Microbiol. 2025 Aug 8;7(8):000991.v3. doi: 10.1099/acmi.0.000991.v3 (PMC12451302; doi:10.1099/acmi.0.000991.v3)
Supplement: Uncited Supplementary Material 1. [file acmi-7-00991-s001.pdf]

SUPPLEMENTARY MATERIAL

AT-HOME, SELF-SAMPLING OF THE SKIN MICROBIOME: DEVELOPMENT OF AN UNSUPERVISED SAMPLING APPROACH

Leng, J., Tyson-Carr, J., Adams, S., Scott, M., Thomas, A., Giesbrecht, T., Fallon, N., Murphy, B., Hoptroff, M., Roberts, C., Paterson, S.

Supplementary Item 1: Unsupervised swabbing instruction given to study participants

Swabbing instructions for obtaining skin samples

Thank you for agreeing to participate in this study. For this study we are requesting that you provide swab samples from the following areas:

- <u>either</u> left or right underarm
- <u>either</u> left or right inner forearm
- <u>either</u> left or right cheek
- the scalp at the top of the head

The same site or side that was swabbed in the Consumer Science Centre will need to be swabbed at home. We have circled the side you sampled in the centre above.

Your pack should contain the following:

- Subject information form
- 1 bag containing 4 swabs and 4 pre-labelled collection tubes one for each of the following:
    - for underarm, forearm, cheek and scalp
- A return envelope

Please do not wash the sampling sites 6 hours prior to collecting the samples. Swabs should be taken at the same time of the day they were taken when visiting the Consumer Science Centre.

Before attempting to take any samples please read these instructions & the additional notes in full.

Warnings and precautions:

- Collection tubes should remain sealed until used to prevent contamination.

- Ensure the swab tip does NOT come into contact with any surface other than the area to be sampled prior to or after collection.
- Please store all items at room temperature.

<u>Instructions for sampling</u>

1. Remove clothing to expose the areas to be sampled. Please swab near to the site on the same side of your body that was swabbed in the Consumer Science Centre. Select the correct tube for the area to be sampled. The label on each tube will include the name of the area being swabbed.

2. Open the swab tube at the red end by pulling and twisting at the same time. **IMPORTANT:** DO NOT touch the swab tip or place it in contact with any surfaces. DO NOT hold the swab between the dented point and the swab tip.

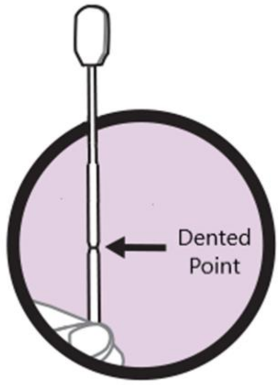

3. To ensure a successful sample collection:

   - Hold the swab so that the **<u>side</u>** of the swab tip is in contact with the skin (rather than the end).
   - Apply continuous pressure on the swab tip.
   - Rub firmly, back and forth for one minute on each site, covering an area approximately the size of a 50 pence piece.
   - Try to keep the swab tip in physical contact with the skin at all times.
   - Continuously rotate the plastic shaft of the swab so that all sides of the swab come into contact with the skin.

IMPORTANT: The swab will bend during this step but will **NOT** break.

4. Remove the lid from the collection tube and place the swab into the collection tube. Hold the tube by the label and bend the swab to break the shaft at the red, dented point (see below). The rest of the swab that has not been put into the tube can be discarded.

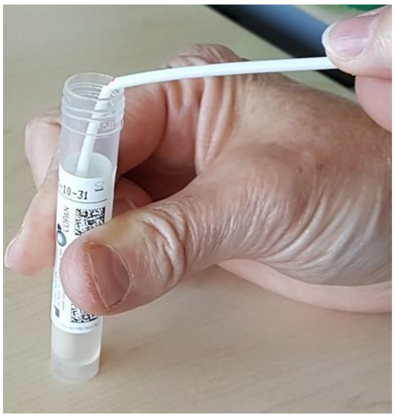

5. Replace the cap and screw the lid tightly to close.

6. Repeat steps 2-6 at the other areas (body sites) using a new, fresh swab for each site and the corresponding collection tube.

7. Place all 4 tubes into the clear bag provided. Place this bag along with the completed Information Sheet back into the envelope and seal it.

8. Return the envelope to the Consumer Centre at your next DEO visit.

Thank you for your co-operation.

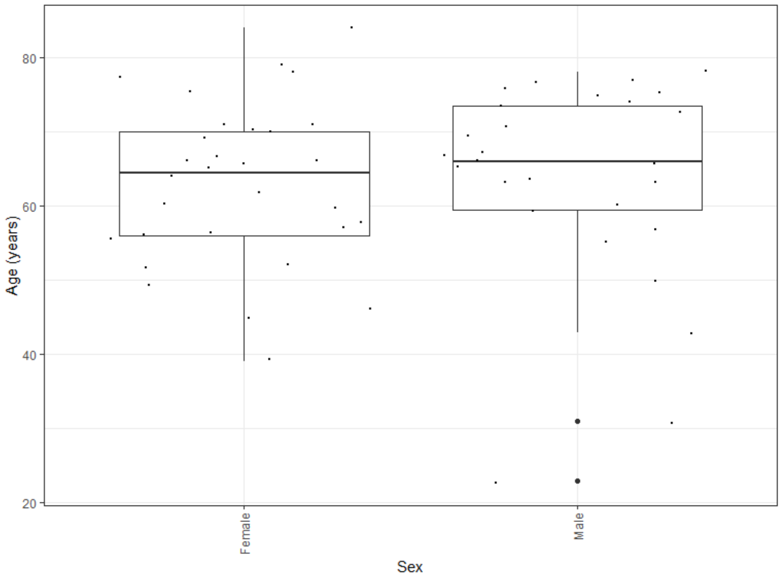

Supplementary item 2: Boxplot showing the range of ages of the study participants.

Supplementary item 3: Number of samples per body site that failed quality control and those within a range of number of reads. Samples that failed and those with less than 10,000 reads were not carried forward for further analyses.

Number of sequencing reads	Axilla	Cheek	Forearm	Scalp	Total
Failed quality control	1	17	44	25	87
< 10,000	0	6	6	4	16
10,000 – 20,000	0	3	10	1	14
20,000 - 30,000	1	2	3	6	12
30,000 - 40,000	9	7	5	4	25
40,000 – 50,000	12	12	6	10	40
50,000 – 60,000	33	20	7	18	78
60,000 - 70,000	25	20	12	13	70
70,000 – 80,000	13	7	12	15	47
80,000 - 90,000	7	8	3	9	27
90,000 – 100,000	5	6	1	2	14
> 100,00	6	4	4	4	18
Total carried forward for further analyses	111	89	62	83	345

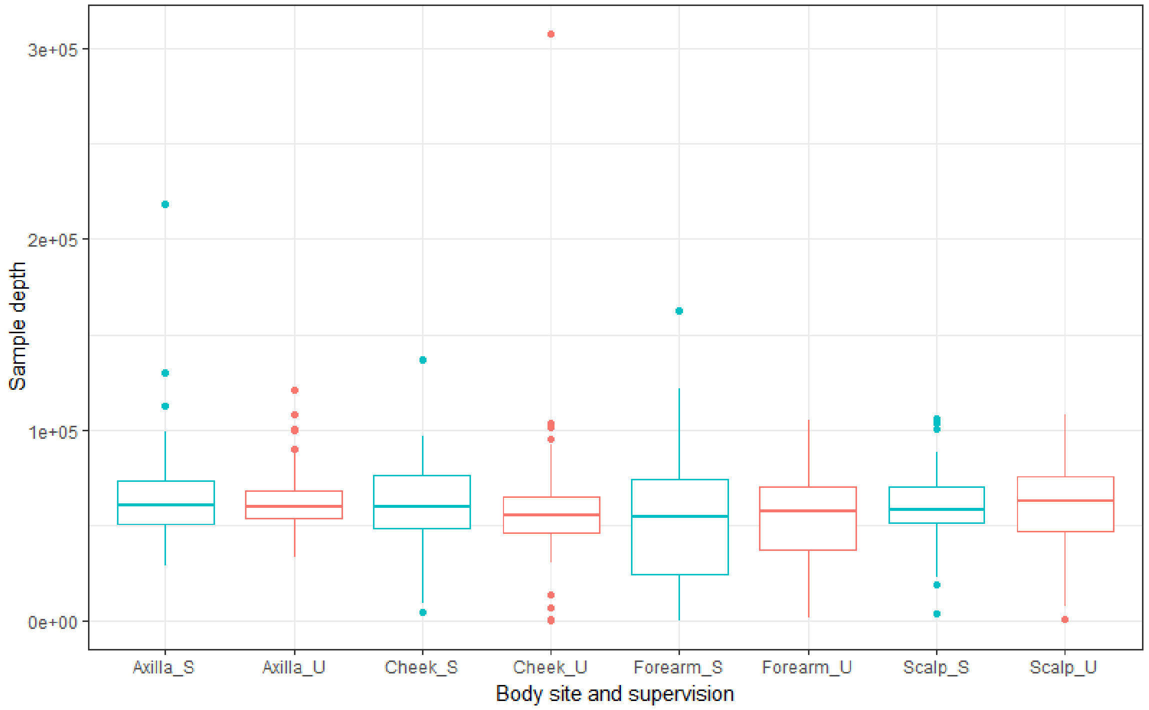

Supplementary item 4: Boxplot of sequencing read numbers when grouped by both body site and supervision (S, supervised and U, unsupervised).

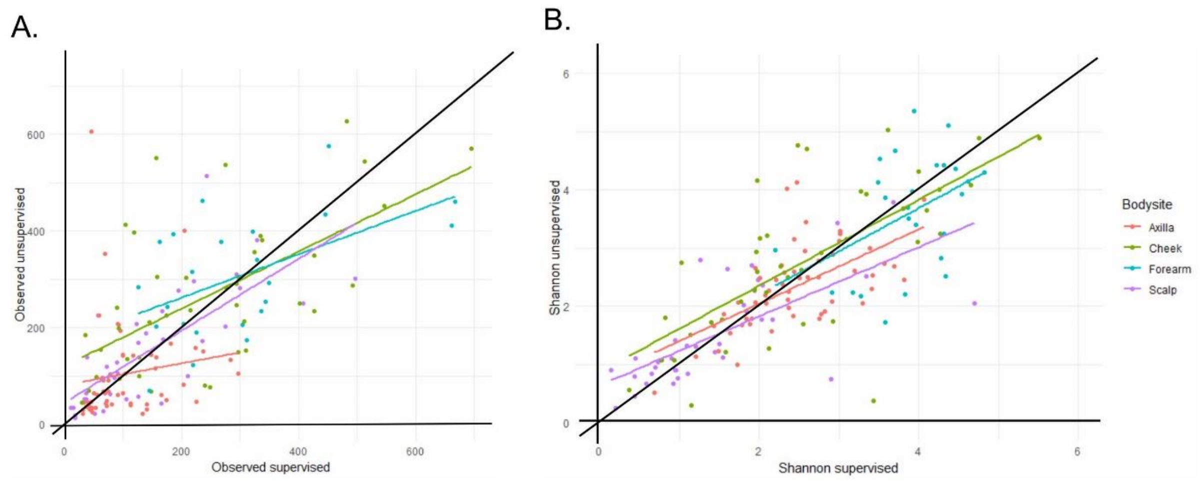

Supplementary item 5: Scatter graphs visualising the A) Observed and B) Shannon diversity of the samples taken with and without supervision. Points are coloured by body sites and the coloured regression lines show the relationship for each of the four body sites sampled. The black line is a reference maker for the perfect correlation (where x = y).

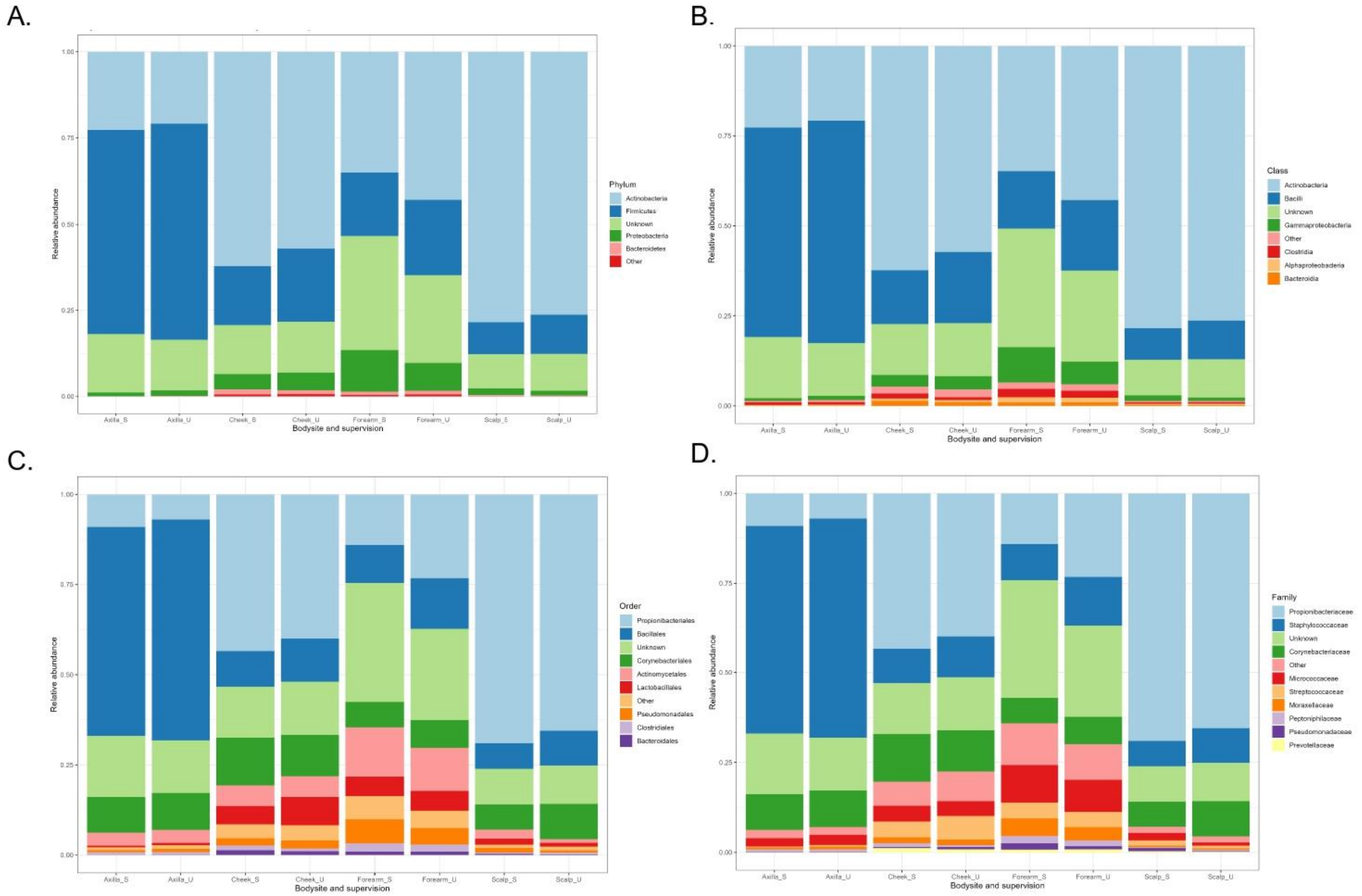

Supplementary item 6: Mean relative abundance of the most prevalent bacterial A) phyla, B) classes, C) orders and D) families as a mean of samples taken supervised (S) and unsupervised (U), for each of the four body sites. All other bacterial genera identified were grouped into “Other”.

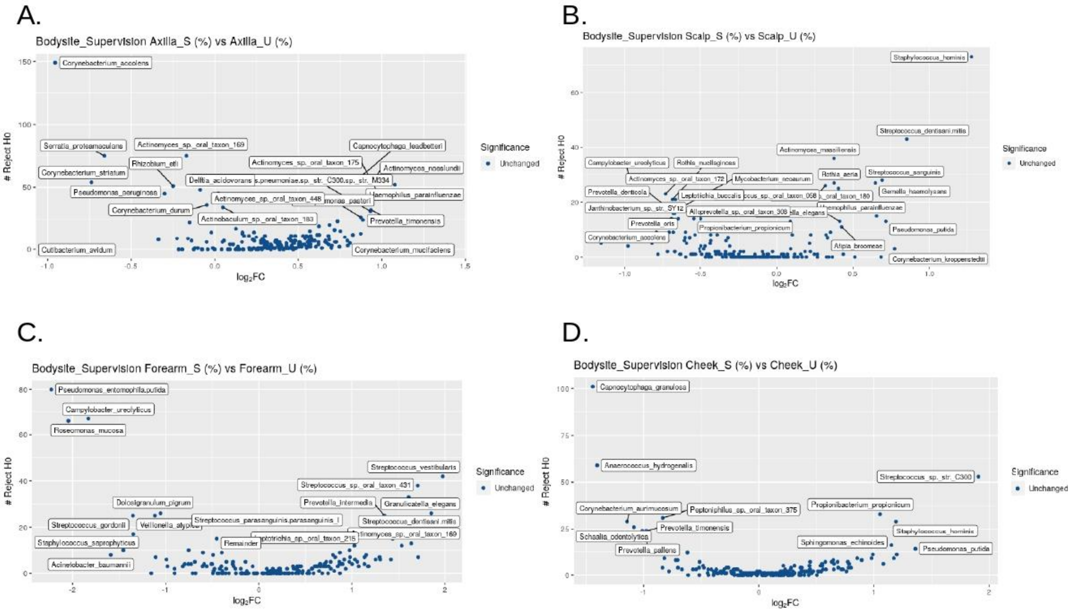

Supplementary item 7: Volcano plots from fastANCOM analysis using relative abundance data comparing samples taken supervised and unsupervised from each of the body sites: A) Axilla, B) scalp, C) Forearm and D) Cheek. No bacterial species were significantly different in abundance between supervised and unsupervised samples as all returned non-significant discovery rates (Q > 0.1). Reject H0 indicates whether the null hypothesis can be rejected, with higher values indicating it can be rejected.
